# Bio-Inspired Optimization-Based Path Planning Algorithms in Unmanned Aerial Vehicles: A Survey

**DOI:** 10.3390/s23063051

**Published:** 2023-03-12

**Authors:** Sabitri Poudel, Muhammad Yeasir Arafat, Sangman Moh

**Affiliations:** Department of Computer Engineering, Chosun University, 309 Pilmun-daero, Dong-gu, Gwangju 61452, Republic of Korea

**Keywords:** bio-inspired algorithm, optimization algorithm, path planning, unmanned aerial vehicle, UAV communication, UAV path planning

## Abstract

Advancements in electronics and software have enabled the rapid development of unmanned aerial vehicles (UAVs) and UAV-assisted applications. Although the mobility of UAVs allows for flexible deployment of networks, it introduces challenges regarding throughput, delay, cost, and energy. Therefore, path planning is an important aspect of UAV communications. Bio-inspired algorithms rely on the inspiration and principles of the biological evolution of nature to achieve robust survival techniques. However, the issues have many nonlinear constraints, which pose a number of problems such as time restrictions and high dimensionality. Recent trends tend to employ bio-inspired optimization algorithms, which are a potential method for handling difficult optimization problems, to address the issues associated with standard optimization algorithms. Focusing on these points, we investigate various bio-inspired algorithms for UAV path planning over the past decade. To the best of our knowledge, no survey on existing bio-inspired algorithms for UAV path planning has been reported in the literature. In this study, we investigate the prevailing bio-inspired algorithms extensively from the perspective of key features, working principles, advantages, and limitations. Subsequently, path planning algorithms are compared with each other in terms of their major features, characteristics, and performance factors. Furthermore, the challenges and future research trends in UAV path planning are summarized and discussed.

## 1. Introduction

Unmanned aerial vehicles (UAVs) are flourishing because of growing demands and technological advancements. Owing to their versatility, flexibility, easy installation, and moderately low operational costs, UAVs have been incorporated into many civilian applications, such as surveillance [1], monitoring [2], emergency communication [3], rescue and disaster management [4], traffic control [5], parcel delivery [6], tracking tasks [7], data gathering [8], pandemic outbreaks [9], and wildfire detection [10]. Path planning in UAVs is one of the most relevant problems. Path planning intends to find the optimal and shortest path and ensure that UAVs operate in a collision-free environment. The use of path planning techniques is essential for computing a safe route in the shortest time. Path planning in UAVs has become a crucial problem in establishing an optimal path between source and destination [11]. Much research in the literature is highly focused on path planning for UAV operations. However, different issues, such as target location and identification, remain when considering the high maneuverability of UAVs. The path planning of UAVs must consider the most precise decisions to achieve several mission-critical tasks.

The scientific community has recently seen a deluge of material on the topic of replicating social and behavioral tendencies. Nowadays, intelligence-based computational techniques are used for information processing, decision-making, and attaining optimization goals across a wide range of science and engineering applications. Numerous methods and algorithms have been created during the past few decades in a variety of domains, including genetic algorithms, artificial neural networks, evolutionary algorithms, and fuzzy algorithms. In the upcoming years, it is anticipated that intelligent optimization algorithms will be more successful at resolving many issues in the fields of engineering, science, medicine, space exploration, and artificial satellites for anomaly and failure detection.

Bio-inspired algorithms, which are short for biologically inspired algorithms, are stochastic and metaheuristic search algorithms from a computational perspective. They are beneficial for solving distributed and multimodal optimization problems [12]. The search procedure used by bio-inspired algorithms is robust and can efficiently maintain diversity. Bio-inspired algorithms can avoid local optimal convergence and achieve globally optimal solutions with higher probabilities [13]. However, with these methods, the injected exploration noises are uncontrolled. As a result, they arrive at a solution, but its universality cannot be guaranteed. By injecting noise into the action space, bio-inspired approaches typically engage in exploratory behavior. One approach is to directly include noise in the agent’s parameters; this can result in more reliable exploration and a wider range of behaviors [14]. Additionally, bio-inspired algorithms allow agents to interact and use feedback mechanisms to resolve various dynamics in a cooperative manner.

Bio-inspired algorithms are commonly used in path optimization algorithms for UAVs owing to their several benefits. The first advantage of bio-inspired algorithms is that they are robust even under adverse conditions because they have self-organized patterns [15]. Second, they can readily adapt to dynamic environments [16,17]. Path planning, trajectory generation, and control systems are major concerns in UAV automation. These terms are often used interchangeably; however, there are some distinctions among them, which are shown in Table 1. Typically, the geometric path component is viewed as path planning, whereas a trajectory is created from a geometric path considering joint limits, velocity, and acceleration constraints.

Recently, various bio-inspired algorithms have been widely used for path planning, target tracking, heterogeneous cooperative control, formation control, routing, and navigation in UAVs [18]. The goal of the optimization process is to identify the best solution to a given issue. Selecting an appropriate algorithm is a crucial step in accomplishing this goal. However, certain issues are so complicated that finding every potential solution might be challenging. Numerous metaheuristic algorithms have been created and published in the literature to simulate the biological behavior of groups of animals or insects by specifying deterministic or random rules to be used in addressing various optimization problems. Different bio-inspired algorithms have been employed to solve the path planning and optimization issues prevailing in UAVs. The bio-inspired algorithms frequently employ a swarm of several cooperative agents to produce the search motions in the search space. Global optimizers are frequently straightforward, adaptable, and unexpectedly effective. Over the past three decades, there have been substantial advancements and the emergence of numerous applications. In this study, we evaluate state-of-the-art bio-inspired path-optimization algorithms proposed for autonomous UAV operations.

Bio-inspired algorithms are also a subset of evolutionary algorithms. Evolutionary algorithms are a group of optimization algorithms that are inspired by the principles of natural selection and evolution [19]. These algorithms use various techniques, such as mutation, crossover, and selection, to simulate the process of evolution and find near-optimal solutions to complex problems. In other words, bio-inspired algorithms are a type of evolutionary algorithm that is specifically designed to mimic the processes and behaviors observed in biological systems. The algorithms are used to solve problems in a variety of fields, including machine learning, computer science, and engineering.

Although bio-inspired optimization approaches have found applications in various fields, there has been no rigorous analysis of their path planning capabilities. By imitating the self-organizing behaviors of biological creatures, bio-inspired optimization algorithms have been demonstrated to be promising methods for highly complicated problems [20]. Therefore, bio-inspired algorithms have attracted the attention of researchers worldwide. UAVs could be rapidly deployed as they are employed to extend the range of communication, make effective use of network data communication competence using UAVs as relays, collect data from large networks in inaccessible or harsh atmospheres, and aid node localization in mobile networks. Most bio-inspired algorithms show better performance in terms of the convergence rate [21].

The main objective of this study was to epitomize the different path planning techniques used for UAV communication. We explored different bio-inspired path planning practices through a precarious investigation of state-of-the-art proposals. In addition, various parameters were considered in the analysis of path planning approaches. These parameters include the length of flight, optimality of the algorithms, cost-effectiveness, time effectiveness, energy effectiveness, collision avoidance, and robustness.

### 1.1. Related Surveys

In the literature, there are surveys on different topics of UAV communications. An overview of trajectory planning, resource allocation, and cooperative UAVs is provided in [22]. In [23], a comprehensive survey of the UAV network characteristics, routing protocols, and seamless handover techniques is provided. The authors summarized the characteristics of UAVs and explored various channel models in [24]. In [25], UAV cooperation techniques were discussed. Various UAV applications in wireless sensor networks (WSNs) were studied in [26]. UAV path planning techniques were discussed and analyzed in [27]. In [28], an extensive review of the optimization techniques used in UAV path planning is provided.

However, several articles have addressed communication problems in UAV networks [29,30,31] and UAV path planning problems, which typically study path planning separately from UAV networks. Path planning for UAVs or swarms of UAVs is one of the most challenging tasks during data collection. From the literature, we established that bio-inspired algorithms are widely used for UAV path planning. Although there have been some surveys on UAV path planning in the literature, no survey on bio-inspired algorithms for UAV path planning has been reported thus far. Therefore, we provide an extensive survey of existing bio-inspired-based UAV path planning techniques. Table 2 summarizes the existing studies on various UAV and UAV-based application topics to confirm the distinctness of our work.

### 1.2. Contributions of This Study

Bio-inspired algorithms frequently employ a swarm of several cooperative agents to produce the search motions in the search space. Global optimizers are frequently straightforward, adaptable, and unexpectedly effective. Over the past three decades, there have been substantial advancements in technologies and the emergence of numerous applications. In this study, we extensively investigated state-of-the-art bio-inspired approaches for UAV path planning. Our contributions can be summarized as follows:

- We provide a brief overview of path planning and address the design issues associated with UAV path planning. Additionally, we present a classification of bio-inspired algorithms for UAV path planning.
- We review various bio-inspired algorithms in the context of UAV path planning. The algorithms are compared in terms of their benefits and limitations, and some promising relevant extensions are provided. Several comparative tables, including various system and performance parameters, are established and discussed.
- Moreover, we discuss challenging issues and future research directions for designing and implementing bio-inspired algorithms for UAV path planning, which can be helpful to researchers in this domain.

### 1.3. Organization of This Paper

This survey is divided into six sections. A preliminary study on UAV path planning in the context of an overview of UAV path planning is presented in Section 2. We also provide design support for UAV path planning. In Section 3, we categorize the bio-inspired algorithms into several categories in the context of UAV path planning. In Section 4, we compare UAV path planning in terms of its advantages and limitations, and some promising relevant extensions are included in the comparison tables. In Section 5, we provide future research guidelines and discuss open issues. Finally, the conclusions of this study are presented in Section 6.

## 2. Preliminaries

The characteristics of UAVs include their size, payload, coverage range, battery life, altitude, and flying principles [32]. Depending on their physical structure, UAVs can be classified as fixed- or rotary-wing UAVs. There are several noteworthy features, such as high flight speeds, a large payload capacity, and a long-lasting battery backup. However, most fixed-wing UAVs lack vertical takeoff and landing (VTOL) capabilities [33]. Owing to their unique physical characteristics, such as their ability to support stationary positions and VTOL capability, rotary-wing UAVs have been widely used in various civilian applications.

### 2.1. Optimization Algorithms

A measurement database of sunshine duration for Algeria’s southern region is provided in [34]. In addition, this study aims to evaluate whether computation models can be used to predict durations. Based on a hybrid model that combines the gray wolf and stochastic fractal search optimization algorithms (GWO-SFS) with an ensemble of random forest regressors, the study proposes a novel optimization algorithm. In [35], the effectiveness of a novel metaheuristic algorithm called dipper throated optimization (DTO) is evaluated in comparison to other optimization techniques. The DTO was tested on seven unimodal benchmark functions and used for feature selection in real-world problems, with results indicating that it outperformed all other algorithms.

A voting classifier that can identify and minimize operational risks in supply chain 4.0 was proposed in [36], which is the fourth industrial revolution’s way of managing the supply chain. The proposed algorithm utilizes a sine cosine dynamic group (SCDG) algorithm and makes adjustments to it in order to be more effective at identifying risks. The proposed approach has a number of advantages. A new machine learning algorithm, called the adaptive dynamic particle swarm algorithm, which was combined with a guided whale optimization algorithm for wind speed ensemble forecasting was studied [37]. This algorithm was designed to improve the accuracy and reliability of predictions made about wind speed and power production in systems with significant wind penetration. An advanced meta-heuristic optimization algorithm for antenna architecture design was presented in [38]. This algorithm combines the sine cosine algorithm and grey wolf optimizer to train a multilayer perceptron. The proposed optimization algorithm provides an objective evaluation of the results obtained and offers accuracy in verifying the procedures’ accuracy. It also offers superior performance and validation stability evaluation of the predicted results to verify the accuracy of the procedures.

### 2.2. Objective of UAV Path Planning

Because UAVs are mobile, their states can be dynamically adjusted to meet the requirements of a particular mission. Moreover, the operation of UAVs can be hampered by obstacles that may exist in dynamic environments. Therefore, UAVs must be autonomous to perform various operations. Owing to this mechanism, UAVs can make their own decisions based on the information provided to them. When planning a path, it is imperative that considerable environmental information be used to ensure believability, safety, and optimality. To avoid threats and obstacles, it is critical to consider constraints, including navigation accuracy, platform maneuverability, arrival time, and fuel power restrictions. The information can be captured using sensors, cameras, and a global positioning system (GPS). UAVs can maximize their performance and efficiency by exploring all decisions using GPS, cameras, and various sensors [39]. Mathematical models have been developed to describe specific tasks, set constraints, and optimize methods for UAV path planning.

### 2.3. Overview of UAV Path Planning

The path planning task involves determining an optimal path for UAVs from an initial point to a target point. All collisions with surrounding obstacles should be avoided during the path determination for UAVs. The determinations should satisfy the physical and kinematic constraints of the UAV, such as electrical energy and kinetic energy [40]. Motion planning, trajectory planning, and navigation are key parts of UAV path planning. Motion planning is critical for UAVs. By turning a crank in the path planning motion, this planning satisfies constraints such as the flight path. Through this technique, the path is optimized in terms of short path length and minimum turning angle. The concept of trajectory planning pertains to motion planning. Path planning involves factoring in the velocity, time, and kinematics of the UAV’s motion. A navigation system comprises several components, including motion planning, trajectory planning, collision avoidance, and localization. In general, it refers to the control and monitoring of the movement of a UAV from one location to another. Autonomy and flight stabilization accuracy are crucial in current UAVs. Navigation systems and their supporting subsystems are critical components of autonomous UAVs. The navigation system uses information from various sensors to estimate the position, velocity, and orientation of the UAV.

### 2.4. Elements of Path Optimization Algorithms

#### 2.4.1. Cost Function

The cost function should be minimized in the optimization process. The cost function for path planning algorithms is basically determined by the path length, threat cost, and energy consumption, and is given as

(1) $$\underset{\Theta ,\varepsilon_{c},\;\tau_{c,\;}}{min}\;a_{1}\cdot f\left(\Theta \right)+a_{2}\cdot f\left(\varepsilon_{c}\right)+a_{3}\cdot f\left(\tau_{c}\right),$$

where $f\left(\Theta \right)$ is path length function, $f\left(\varepsilon_{c}\right)$ is energy consumption function, and $f\left(\tau_{c}\right)$ is threat function. $a_{1}$, $a_{2}$, and $a_{3}$ are positive path constraints and $0\leq (a_{1},\;a_{2},\;a_{3})\leq 1$.

#### 2.4.2. Flight Constraints

During the flight mission, the UAV must abide by its dynamic constraints when planning its path. Considering some common waypoints to demonstrate the flight constraints in UAVs, we assume that path coordinates at t−1, t, and t+1 are$\;\left(X_{i-1},Y_{i-1},Z_{i-1}\right)$, $\left(X_{i},Y_{i},Z_{i}\right)$, and $\left(X_{i+1},Y_{i+1},Z_{i+1}\right)$, respectively. Other flight constraints are defined as the UAV’s roll, pitch, and yaw orientations, respectively. Flight height restrictions are determined by the particulars of the UAV flight mission, not only to minimize energy usage but also to provide a flexible path.

#### 2.4.3. Collision Detection and Avoidance

UAVs are embedded with different sensors that help them detect dynamic and static objects during their flight. Obstacle detection and avoidance play significant roles in an efficient path planning process. The basic steps involved during collision detection and avoidance are a sequence of observe/sense, identify the potential risks, compute new path coordinates, take new path coordinates to maneuver, and continue maneuvering.

### 2.5. Design Issues in UAV Path Planning

**Figure 1:**
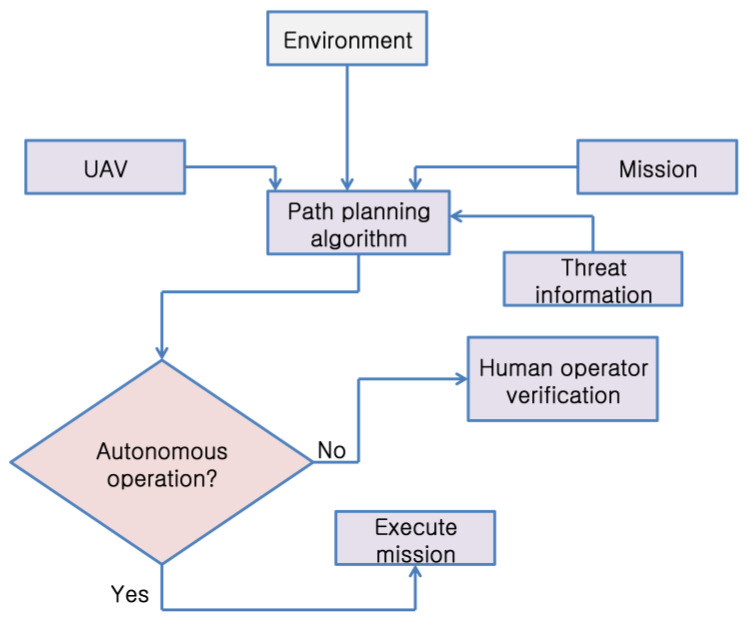
Constraints on path planning in UAVs.

Many research studies have been conducted to resolve the path planning issues of UAVs in terms of complexity. In [41,42], the authors described different areas using different sweep courses to identify the best path. In addition, the use of back-and-forth arrangements to reduce the distance between sub-areas was further studied in [43]. Collisions and forbidden areas were outlined in [44]. In [45,46], the authors proposed a hybrid decomposition method and estimated a cellular decomposition method for dividing the coverage area into triangular shapes. A spiral design was proposed in [47] to facilitate path planning in areas with complex coverage. Planning UAV paths primarily involves minimizing the computation time and cost for the best possible path coordinates. The path coordinates obtained should likewise be energy-efficient, time-efficient, and collision-free. Simultaneously, the design must be robust and comprehensive in path planning practices. The main design considerations for UAV path planning are discussed in the following sections. The different constraints that must be considered while designing the path-optimization algorithms are depicted in Figure 1.

#### 2.5.1. Path Length

Most UAV applications target emergency or critical environments. The path of a UAV plays a crucial role in determining its energy and time consumption and represents the entire distance traveled by the UAV from the start to the end points. The UAV path should be as short as possible to achieve high network performance.

#### 2.5.2. Optimality

A path planning algorithm must be time-efficient, cost-effective, and energy-efficient. Planning a UAV path involves three major aspects: cost, time, and energy.

#### 2.5.3. Extensiveness

To discover a path, it is necessary to consider the completeness of the algorithm during path planning. The platform is equipped with UAV technology and helps to find an ideal solution.

#### 2.5.4. Computation Time and Cost

The path planning algorithms are supposed to be time-efficient if they can accomplish a task in the shortest amount of time while dealing with obstacles. UAVs should find the optimum path for completing the intended operation.

The entire computational cost of the UAV network determines whether the proposed algorithm is cost-efficient. Numerous factors, such as the cost of the nodes, cost of fuel, cost of the battery, cost of memory, as well as cost of the software and hardware used in UAVs, are considered.

#### 2.5.5. Energy Efficiency

The path planning algorithm for UAVs should be energy-efficient because UAVs are power-constrained devices, and the energy of UAVs determines the network lifetime. If UAVs use less fuel, fewer batteries, and a smooth path to reach their targets, their energy cost is minimized. UAVs use a limited amount of energy. To ensure a safe return to the intended destination, it is essential to ensure that the UAV completes its mission and returns before its energy is consumed.

#### 2.5.6. Robustness

The path planning algorithm must be sufficiently robust to withstand location-sensitive device malfunctions as well as other errors caused by environmental factors during the operation of a mission.

#### 2.5.7. Collision Avoidance

The optimal path must be able to avoid all obstructions encountered during flight. UAVs operate in dynamic and uncertain environments. UAVs should be able to detect other elements in their way to prevent a collision. This is because UAVs can successfully choose other path coordinates so that they can avoid any possible damage.

#### 2.5.8. Prohibited Areas

Generally, UAV flight is prohibited in certain areas, such as military and government offices. UAVs should avoid restricted areas within their trajectories when designing their flight paths. Path coordinates with the lowest possible path cost and excluding prohibited regions must be chosen.

#### 2.5.9. Coverage and Connectivity Constraints

The communication range limits the capabilities of UAVs. In addition to the mobile design of UAV communication systems, many other parameters of the joint system are affected by the communication requirements, including power control, user association, and resource allocation. Communication connections between UAVs must be considered in multi-UAV path planning to avoid collisions. Therefore, the factors have a significant effect on path planning, and they must be considered.

### 2.6. Optimization Objective for UAV Path Planning

The path planning algorithms must be evaluated on the basis of performance indicators. UAVs have a wide range of applications owing to their mobility. Although there are many opportunities for UAV networks, there are also many challenges and problems. For UAV path planning, various optimization factors, such as time, energy, coverage, collision, throughput, quality of service (QoS), and interference management, must be considered.

One of the major factors that limit a UAV’s path is the amount of time taken to travel between the initial and target positions. For UAV communication, it is crucial to minimize flight time. Moreover, compared with a single UAV, multi-UAV communication systems consume more energy, thereby reducing the service life and performance of the UAVs; therefore, it is important to reduce energy consumption. To provide hotspot coverage, the UAV must choose an efficient flight path that allows it to access all areas within the plan [48,49]. To ensure safety, conflicts and collisions between multiple UAVs should be avoided. Typically, the throughput of a UAV is determined by the maximum rate of data reception and transmission. To accomplish the task, UAVs are required to transmit information within a specified period in order to complete the total throughput. Notably, UAV paths have a significant influence on QoS in UAV communication [50]. To guarantee an adequate QoS, the precise path of each UAV must be carefully considered. To reduce the impact of interference, an interference management model must be established, and an effective solution must be developed for interference events. Because UAVs share spectrum resources, interference issues can occur in multi-UAV environments. UAVs could generate a greater amount of interference from adjacent cells in the uplink while experiencing a greater amount of interference from the downlink [51].

## 3. Bio-Inspired Algorithms for UAV Path Planning

Behavioral neuroscience and physiological biology are the two fields of biological behavior that use bio-inspired models. In the model, path planning techniques are used to manage and construct a possible solution within a complex UAV environment. Specifically, the model proposes a stable method that converges to a goal. The model is composed of various algorithms that utilize evolutionary techniques and neural networks to describe the path planning techniques used by UAVs [52].

**Figure 2:**
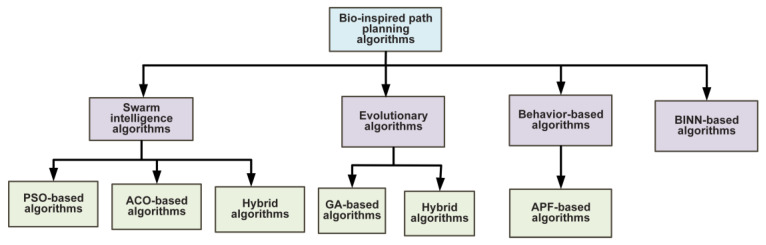
Classification hierarchy of bio-inspired path planning algorithms for UAVs.

Bio-inspired algorithms mimic biological behaviors based on their ability to analyze problems. Instead of building complicated environmental models, these algorithms recommend using a strong search algorithm. Bio-inspired algorithms are categorized into swarm intelligence, evolutionary, behavior-based, and multi-fusion-based algorithms. Figure 2 provides a detailed classification hierarchy of the bio-inspired algorithms for UAV path planning.

### 3.1. Swarm Intelligence Algorithms

In recent years, several bio-inspired algorithms have been proposed and applied to path planning. It is imperative to consider several indicators when designing a fitness function, including energy consumption, quality of service, and coverage. Using swarm intelligence, the path planning problem consists of searching for all possible paths from the starting point to the target point to minimize the path’s total cost. Some studies have established multiple objective functions by considering performance indicators as joint constraints, such as the network data rate and coverage. To plan the path, swarm intelligence algorithms have been used to explore various candidate solutions [53]. Optimizing the total cost is the objective of the optimization process. A thorough analysis and design of the objective functions and constraints are required according to various scenarios and tasks. Depending on the application, the path planning parameters should be adjusted. According to the requirements of the application, the designer can select from several trade-off solutions.

Generally, swarm intelligence refers to the cooperative behavior of self-organized and distributed particles. In these algorithms, each particle operates independently, based on a search-centric approach. Their exploration of the environment is further enhanced through collaboration with nearby neighbors. This process has two distinct phases: exploration and search. Through different communication channels, particles detect, verify, and broadcast data to their neighbors, which are then received by other swarm agents. The goal of the search phase is to determine the direction in which each agent should travel by combining its specific data with the data provided by its neighbors. Pigeon-inspired optimization [54], ant colony optimization (ACO) [55], glowworm swarm optimization (GSO) [56], the plant growth route planning algorithm (PGR) [57], and particle swarm optimization (PSO) [58] are commonly used swarm intelligence-based algorithms for path planning in UAVs.

#### 3.1.1. PSO-Based Algorithms

To avoid the PSO local minimum, a time-varying adaptive inertia weight called NPSO was proposed [59] for the PSO, which significantly improves the generation of an optimal UAV path. To address the shortcomings of PSO algorithms, this study improves the weight and learning factor of the particle swarms [60]. An improved PSO (IPSO) algorithm can plan autonomous paths for UAVs, resulting in the optimization of the operating environment for UAVs and aiding applications in related fields. In [61], a distributed particle swarm optimization (DPSO)-based path planning algorithm was presented for UAV swarms during a reconnaissance mission. Targets were organized into clusters, and different tactical concerns were considered during the planning process. In [62], a tri-objective optimization problem was formulated for planning UAV paths that considers the length, height, and tuning angle of the path. A multi-objective PSO algorithm (MO-PSO) was used to solve the tri-objective optimization problem.

#### 3.1.2. ACO-Based Algorithms

Ants’ cooperation through pheromones is the basis of ACO, which is a swarm intelligence algorithm. ACO has superior scalability and robustness, making it compatible with UAV path planning. Using a combination of relative theories and methods concerning mobile delay tolerant networks, the authors presented a feasible algorithm for planning multiple-object UAV ferrying paths by utilizing grid maps and ant colony optimization algorithms (GM-ACO) to accommodate multiple communication objects and information priorities [63]. In [64], ACO was used to build an effective UAV path planning strategy based on the obstacle-avoidance constraint. In [65], UAV path planning to minimize the path cost for information transmission was the focus. A multi-UAV path planning approach that combines the K-means clustering algorithm with an improved max–min ant system (MMAS) is proposed. Using K-means clustering, the authors reduced the size of the problem, allowing for better search efficiency.

#### 3.1.3. Hybrid Algorithms

A hybrid algorithm can be created by combining multiple bio-inspired algorithms. In [21], a hybrid path planning algorithm that combined PSO with the harmony search algorithm (HSA) was proposed. PSO-HSA performs well in terms of path selection, obstacle avoidance, and path length minimization, which reduces the power consumption of the UAV. In [66], a hybrid path planning algorithm based on the bat algorithm (BA) and the artificial bee colony algorithm (ABC) was proposed and is called the improved bat algorithm (IBA). A major purpose of the IBA is to modify the BA using ABC and resolve the problem of its poor local search capability. Grey wolf optimization (GWO) is based on the wolf hierarchy structure. In [67], an adaptive GWO (AGWO) algorithm for UAV path planning was proposed. By controlling the adaptive convergence parameters, AGWO reduces the time required for path planning. Path planning in complex, dynamic environments is challenging. In [68], a culture algorithm (CA) for UAV path planning was used. CA is a double-level algorithm based on human behavior. According to CA, knowledge and experience in the belief space can direct individual evolution in a population space in a positive direction. In [69], a hybrid path planning algorithm, GWFOA, was proposed, based on GWO and the fruit fly optimization (FOA) algorithm. In the proposed approach, GWO is used for the initial path planning, and FOA is then used to avoid the local minimum problem and obtain the optimal solution. In [39], a hybrid path planning (HPP) algorithm for UAV-aided WSNs by combining the probabilistic roadmap (PRM) and ABC algorithms was proposed. The PRM was used to find the shortest path, and the ABC algorithm was used to optimize the path. For mobile robot path planning, an improved sparrow search algorithm (ISSA) was used in [70]. According to the authors, the ISSA can deliver a reliable path in a short amount of time.

### 3.2. Evolutionary Algorithms

**Figure 3:**
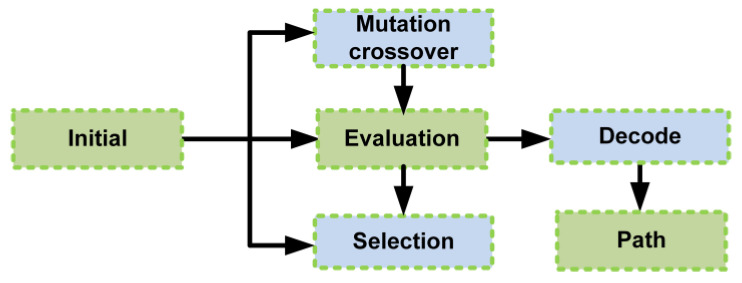
Process of evolutionary algorithms in UAV path planning.

The evolutionary algorithms are exploratory search algorithms that can provide global solutions to complex optimization problems. An evolutionary algorithm increases the probability of exploring near-optimal results during the initial optimization. It does not require fitness gradient information to proceed and can be easily processed in parallel to escape local minima. Evolutionary algorithms are based on natural evolutionary processes, such as reproduction, recombination, mutation, and selection, as shown in Figure 3. These processes were used for optimization, and the fitness function was used to determine the excellence of the obtained results. UAV path planning algorithms use genetic algorithms (GAs), which are widely known evolutionary algorithms.

#### 3.2.1. GA-Based Algorithms

A path planning problem for UAVs was studied in [71]. The GA, simulated annealing (GASA), and targeted mutation (TM) algorithms were used together to solve path planning problems. The proposed GASA enhances the mutation strategy. This algorithm represents a significant improvement over the TM algorithm. By utilizing a random mutation mechanism, it is possible to develop a more effective mutation strategy. As a result, this strategy should be incorporated into the genetic mutation process of the GA algorithm to increase population diversity and search speed. In [72], it was reported that a multi-UAV target search path planning algorithm was developed using K-means and GA. Ref. [73] proposed a novel UAV path planning method based on an immune GA (IGA) algorithm. IGAs introduce immune operators and concentration mechanisms that address the inherent weaknesses of GA, including premature convergence and slow convergence speeds. In [74], the authors proposed a fast genetic algorithm based on a parallel implementation of a GA on graphics processing units for the path planning of fixed-wing UAVs. In [75], a hardware-oriented path planning approach for UAV was proposed, based on an application-specific evolutionary algorithm (ASEA).

#### 3.2.2. Hybrid Algorithms

In [76], the path planning for a fixed-wing UAV in a 3D scenario was studied. To solve the complex UAV path planning problem, the authors developed a hybrid algorithm based on GA and PSO. A deep learning with GA (DL-GA) algorithm was proposed for multi-UAV data collection [77]. The GA collects and processes states and paths from various scenarios and uses them to train deep neural networks to rapidly generate optimized paths within the constraints of familiar scenarios and deadlines. In [78], a hybrid path planning algorithm based on a group search optimizer (GSO) with differential evolution (DE) was proposed. First, GSO was used to update the UAV flight path based on the search angle and distance. Within the search area, the evolutionary process uses self-organization and self-regulation to modify the possible path of the UAV. In [79], a path planning algorithm, differential symbiotic organism search (HDSOS), based on DE and symbiotic organism search (SOS), was proposed. A significant advantage of HDSOS is its ability to preserve the local search capability of SOS while boasting impressive global search capabilities. In [80], a path planning algorithm for dynamic environments based on ACO and DE named MMACO was proposed. To navigate from the initial point to the targeted area from a colony, MMACO upgraded the route and minimized the number of participants. To reach the targeted area as soon as possible, DE determines which sub-colony is most suitable for reaching the region.

### 3.3. Behavior-Based Algorithms

Path planning is a key aspect of UAV operations. Path planning is performed using a behavior-based approach [81]. This method manages complex environments and makes it easy to design and test behaviors.

#### Artificial Potential Field (APF)-Based Algorithms

Dynamic path planning can be simplified and made easier using the APF. A particle represents an object’s environment. As it moves around the configuration space, it is controlled by potential fields. The path of the UAV is calculated based on the resultant fields from the initial point to the target point. However, the conventional APF is affected by local minima, which causes UAVs to become stuck before they reach the target. In [82], an improved APF approach, IAPF, was used to design a collision-free UAV path. The proposed IAPF avoids the local minimum problem during path optimization. Using the Q-learning algorithm and APF approach, the authors devised an effective path planning algorithm for UAVs in [83]. In an unknown dynamic environment, the Q-APF performs well.

### 3.4. Bio-Inspired Neural Network (BINN)-Based Algorithms

A BINN-based approach has been used because of its distinct advantages. That is, it does not require learning and is easy to implement. For UAV path planning in an unknown three-dimensional (3D) environment, BINN exhibits some limitations, such as complex computing problems when the environment is very large and repeated path problems when obstacles are larger than the sensors can detect. In [13], a multi-UAV path planning and tracking (MPPTM) algorithm was proposed. BINN-based coverage planning was proposed in [84]. The authors solved a coverage-planning problem for a fleet of UAVs exploring critical areas. Map coverage, uniform UAV distribution, and collision avoidance between UAVs and obstacles are the three main objectives. In [85], a multi-UAV target-tracking algorithm (MUTT) for unknown environments was proposed, where the target is unpredictable and intelligent. In the bio-inspired neural network, the paths of hunting AUVs are guided, and the results indicate that they can achieve the desired hunting results. A BINN model can be used to model the working environment of the UAVs. In [86], a path planning algorithm based on the sparrow search algorithm (SSA) and BINN was proposed. The algorithm first scans the flight environment and smooths it before increasing it to obtain a safe surface. Subsequently, it uses SSA to determine the nodes with the lowest comprehensive cost on a safe surface. The improved BINN method was used to achieve dynamic obstacle avoidance when a dynamic obstacle was detected on a predetermined trajectory. In [87], an optimal path planning model for UAV surveillance systems based on PSO and an online adaptive neural network (ANN) was proposed. The ANN optimizes the learning rate and minimizes the tracking error.

## 4. Comparison of Bio-Inspired Path Planning Algorithms for UAVs

In this section, we compare different algorithms for bio-inspired UAV path planning. As summarized in Table 3, the bio-inspired algorithms for optimizing UAV path planning are compared in terms of their main ideas, evaluation tools, and design approaches. MATLAB is a widely used simulation tool for developing algorithms for UAV path planning, as listed in Table 3. The hybrid algorithms perform better in terms of algorithm convergence and avoiding the local optimal problem.

In Table 4, bio-inspired path planning algorithms are compared based on their advantages, limitations, and possible enhancements that could be made in the future. Table 4 summarizes the performance-centric advantages and limitations of the bio-inspired path planning algorithm, thereby allowing readers to gain a better understanding of how each algorithm works. Furthermore, we provide suggestions for future improvements to all the path planning algorithms.

The operational features of the algorithms, such as energy, time, complexity, threats, and computation time, are compared in Table 5. From the tabular study, it is apparent that most path planning algorithms focus on finding the shortest, safest, and most efficient paths. Table 5 shows that the simulations of the experiments considered both 2D and static environments. This is despite the fact that the UAV environment is 3D and the obstacles are both static and dynamic.

## 5. Challenges and Future Research Directions

Although UAV technology is an emerging area, there are numerous hurdles to safe and reliable UAV communication. In UAV path planning, most issues stem from the dynamic network of UAVs and the surrounding environment. Furthermore, the performance of an algorithm can be impacted by algorithm-dependent parameters in all bio-inspired algorithms. However, it is unclear which settings or values are appropriate for the parameters in order to attain the best performance. Many bio-inspired path planning algorithms have been proposed for the successful completion of UAV missions in various application areas. Nevertheless, the proposed algorithms still have some limitations when implemented in real-world scenarios. In this section, we discuss some issues and challenges faced by UAV path planning algorithms in association with promising future directions.

### 5.1. Connectivity and Coordination

UAVs are used in different application scenarios. The principal demand that needs to be explored for qualitative UAV operation is connectivity and coordination in UAV networks [88]. A cooperation implementation mechanism should be used for UAVs to collaborate with distributed application environments. Numerous efforts have been made to design error-free mechanisms to guarantee UAV cooperation and coordination. A comprehensive structure, including explanations of various issues associated with UAV communication, such as detection, data gathering, broadcasting, and timely distribution, is in need of improvement for researchers in this field. Considering the different application domains, UAVs need to establish connections with other UAVs, ground nodes, and other infrastructure.

### 5.2. Dynamic Network Topology

UAVs are deployed in dynamic environments that are full of risks and uncertainties. In addition, the high mobility of UAVs is responsible for fluctuations in network and radio channels. Communication is distorted when the information is delayed. Therefore, the network can be intermittent and experience latency, which is unacceptable in UAV applications [89]. UAVs require proper network information to determine the most appropriate and instantaneous communication methods.

### 5.3. Adaptability

For UAV networks, adaptability considers both network adaptability and adaptability to unpredictable mission demands [90]. A UAV network should be able to adapt to changing topologies. In large applications, compliance with network topology variations is a prime requisite because high mobility may result in extremely variable topography and channel conditions. To accomplish the mission effectively, the mission objectives and tasks assigned to UAVs can be modified. Algorithms with high adaptivity features must be designed to accomplish missions.

### 5.4. Power Consumption and Network Lifetime

UAVs are powered by batteries; hence, energy efficiency will always be a prime concern when designing path planning algorithms [91]. Designing an energy-efficient path enhances the network performance of the UAV. The majority of UAV path planning research is concerned primarily with energy minimization; however, UAVs face some challenges that degrade the network lifetime. The methods used to harvest energy from natural sources, such as the sun and wind, can extend the lifetime of a network. However, these resources may not be available continuously. Determining a short and smooth path helps reduce the power consumption of UAVs.

### 5.5. Localization

Owing to their high maneuverability, the locations of UAVs may not be predictable under certain circumstances. However, UAVs require accurate location information within a short time period. In addition, the location of UAVs in the network is crucial for establishing appropriate communication among UAVs. Several localization techniques have been proposed to efficiently locate UAVs within a network. The GPS is widely used for localization purposes. However, the GPS cannot provide accurate position information within a short period of time, which adversely affects UAV performance. It is possible to consider other strategies, such as network-based localization, which relies on the exchange of packets, and localization based on height, to enhance the localization process. Moreover, some studies have focused on fuzzy logic to improve localization accuracy based on a weighted centroid [92].

### 5.6. UAV Speed

With mobile UAV nodes, the resulting network topology is frequently changed. The UAV’s speed depends on the type and size of the UAV. Small UAVs generally fly at a speed of approximately 15 m/s, whereas large UAVs can fly as fast as 150 m/s. The speed of UAVs can be determined on the basis of the requirements of target applications. Furthermore, optimizing path coordinates is a crucial factor that determines the efficiency of energy consumption and spectrum utilization. The tradeoff between speed and agility in UAV networks should be addressed. Emerging technologies can help UAVs extend their endurance. Thus, restrictions caused by speed and acceleration should be considered in field experiments. Optimizing the speed of UAVs can improve the performance of UAV networks [93].

### 5.7. Scalability

A common challenge in UAV networks is UAV operability in both sparse and dense networks. Hence, the protocols in UAV networks should be able to adapt to both scenarios to improve the network’s performance. Furthermore, the number of active UAVs is likely to vary when different factors are considered. The design of scalable protocols has been a significant concern for researchers and developers in this field. The topology of UAVs may change owing to their high mobility and death. Scalability is also a major concern in UAV communication [94]. A cross-layer protocol, in which information is exchanged between layers to establish peer-to-peer connections between UAVs, could be the solution.

### 5.8. Mobility Management

UAVs require definite aerial mobility, which directly influences UAV communication. When UAVs fly at extremely low speeds, they can cover a smaller area, resulting in network delays. In most cases, UAVs are used for disaster management, rescue operations, and surveillance, all of which require low latency. In contrast, if UAVs choose to fly at high speeds, it is very challenging to achieve uninterrupted connectivity between them and other devices. This degrades the overall performance of the UAV-based networks. Mobility management techniques can play an important role in maintaining the accuracy and reliability of topology management in UAV networks [95].

### 5.9. Network Density

Network density significantly affects the UAV’s performance. UAVs can be deployed sparsely or extremely densely, depending on their type, objectives, and missions. If UAVs with a wide range of transmissions and high speeds are used, a smaller number of UAVs can be considered. Otherwise, a large number of UAVs must be deployed to meet network requirements. For any application involving a large number of UAVs, effective collaboration among the deployed UAVs is essential. A path planning algorithm should be adapted according to the number of UAVs in the network [96]. Multiple clustering methods help improve the communication procedure for dense UAV networks. A reliable alternative solution for UAV communication is the design of adjustable density-based protocols. Similarly, flexible and energy-efficient clustering methods can improve the performance of large-scale UAV networks.

### 5.10. Applications

In a specific application, the number of design issues tends to be small or moderate, often under several hundred parameters, despite the wide range of applications for bio-inspired and evolutionary algorithms. The application scenario considered for an algorithm is the most important indicator of how effectively it can solve a wide range of issues [97]. Hence, the application field for a bio-inspired algorithm must be understood beforehand to achieve the mission objectives. Moreover, the algorithm for a given scenario should be properly selected or designed.

### 5.11. Uncertainties

It is anticipated that a path planning algorithm presented with a safety assurance would be able to identify UAV trajectories in the presence of various types of uncertainties. It is necessary to ensure the safe operations of UAVs traveling through challenging settings in order to fully utilize their capabilities. However, a number of uncertainties, such as UAV model uncertainties and location uncertainties, present a significant operational problem. Furthermore, the different types of obstacles that UAVs may encounter during their flight are also uncertainties imposed by their dynamic, complex environments [90]. Due to sensor uncertainties, optimization problems may occasionally be impossible to solve. The uncertainties regarding secure communication are yet another major issue in UAV path planning. UAV-based applications can be vulnerable to various threats if the uncertainties are not sufficiently taken into account. Designing a safety radius can be a solution to avoid the risks of collisions [98,99,100,101,102].

### 5.12. Optimality

Path planning algorithms usually aim to achieve the optimal path, which refers to the ideal path that minimizes objective optimization functions. Bio-inspired algorithms are model-free approaches and, hence, cannot result in the optimal solution. However, they can find near-to-optimal results if some model-based planners are considered [102]. Modeling and controlling approaches can significantly reduce the amount of computing needed to solve high-dimensional path planning problems. Four conditions must be satisfied for a path planning algorithm to be optimal. First, the path planning approach must always be able to determine the appropriate path in real-world static situations. Second, the approach also needs to be able to change with the environment. Third, it must support and deepen the self-referencing technique that was chosen. Finally, it needs to be as simple as possible in terms of complexity, data storage, and processing time.

## 6. Conclusions

Numerous real-world applications involve the optimization of specific goals, such as cost minimization, energy minimization, performance enhancement, and sustainability. Bio-inspired algorithms are currently popular due to their adaptability and efficiency. In this study, we presented an extensive survey of bio-inspired path planning approaches for UAVs. The bio-inspired UAV path planning algorithms were classified into four groups: swarm intelligence, evolutionary, behavior-based, and BINN-based algorithms. Each path planning algorithm was examined in terms of its design ideologies and operational features. Then, the algorithms were compared with each other in terms of their unique features, advantages, and limitations. By comparing the investigated algorithms, one can easily acquire ideas and options for selecting an existing path planning optimization or for proposing a novel one for a specific application. Some of the exposed issues and challenges that will assist researchers and developers were also discussed. There are still many possibilities for designing and optimizing more efficient path planning algorithms for various UAV network applications.

## Figures and Tables

**Table 1:** Comparison of path planning, trajectory planning, and control systems.

Category	Definition	Type	Model
Path planning	Generates a geometric path from a starting point to end, passing through pre-identified points in the operating space	Heuristic search methods and intelligent algorithms	Model-free
Trajectory planning	Finds a time series of successive joint angles that allows moving a robot from a starting configuration towards a goal configuration in order to achieve a task	Space–time paths, successions of actions, and past–future arcs	Model dependent
Control systems	Optimal control is a process of determining control and state inputs for a system over a time period to minimize a cost function	Manual, semiautomatic or arcade, and automatic	Model dependent

**Table 2:** Summary of existing surveys.

Ref.	Year	Description	Challenges	Collision Avoidance	Flight Control	Path Plan	Bio-Inspired Approach	Application	Design Issues
[22]	2021	Reviews AI-enabled routing protocols for UAVs	Yes	No	No	No	Yes	Yes	No
[23]	2015	Studies cluster-based routing protocols in wireless networks	Yes	No	No	No	No	No	Yes
[24]	2019	Studies different air-to-ground propagation channels for UAVs	Yes	No	No	No	Yes	Yes	Yes
[27]	2020	Reviews path planning techniques in UAVs	Yes	Yes	No	Yes	No	Yes	No
[28]	2022	Reviews optimization methods for motion planning in UAVs	Yes	Yes	No	No	Yes	No	Yes
[29]	2020	Reviews medium-access control protocols for UAVs	Yes	Yes	No	No	No	No	Yes
Our work	2023	Studies different bio-inspired approaches considered for UAV path planning	Yes	Yes	Yes	Yes	Yes	Yes	Yes

**Table 3:** Comparison of the bio-inspired algorithms in terms of main theme, tool, and design approaches.

Type	Category	Algorithm	Main Theme	Simulation Tool	Algorithm Used
Swarm intelligence algorithms	PSO-based algorithms	NPSO [59]	Analyzes numerous inertia weights anticipated for PSO to advance the particle diversity.	Python	Modified PSO
		IPSO [60]	Provides self-directed routes for UAVs in a cost-effective and efficient manner, enhances the environment in which UAVs operate, and aids UAV-assisted applications.	MATLAB	PSO
		DPSO [61]	The DPSO algorithm is used to plan paths in which targets are clustered, and a real UAV is correlated for each particle.	Monte-Carlo	Maximum density convergence DPSO
		MO-PSO [62]	For enhancing the efficiency of the algorithm, a vibration function is introduced to the colliding solutions.	MATLAB	Multi-objective PSO
	ACO-based algorithms	GM-ACO [63]	A method for communicating with multiple UAVs using a synergetic path plan.	MATLAB	ACO
		ACO [64]	The shortest UAV route selection and obstacle avoidance during flight.	Experimental and numerical formulation	ACO
		MMAC [65]	K-means clustering algorithm and improved max–min ACO-based path planning for multi-UAVs.	–	K-means clustering and PSO
	Hybrid algorithms	PSO-HSA [21]	A hybrid algorithm that performs both exploratory and exploitative searches.	MATLAB	PSO and HSA
		IBA [66]	Finds the shortest and safest path in complex, 3D battleground environments.	MATLAB	BA integrated into ABC algorithm
		AGWO [67]	Updates the position of individuals by adjusting the convergence factor and an adaptive weight factor.	Equivalent 3D digital map	GWO
		CA [68]	CA-based path planning using situational and normative knowledge in dynamic environments.	Experimental analysis	CA
		GWFOA [69]	Planning UAV paths for oilfield inspections.	MATLAB	GWO and FOA
		HPP [39]	Assures a short, efficient, and collision-free trajectory for UAV-based emergency situations.	MATLAB	PRM and ABC
		ISSA [70]	Inspired by the group wisdom, searching, and anti-predation actions of sparrows, the algorithm produces high-quality paths with fast convergence.	MATLAB	SSA
Evolutionary algorithms	GA-based algorithms	GASA [71]	DE mutation in GA is introduced to increase the diversity of algorithm mutations.	–	GA
		GA [72]	Solving the problem of path planning in multi-UAV-based target searches.	Java	GA and K-means
		IGA [73]	Improves the inherent shortcomings of the untimely and slow speed of convergence prevailing in GA.	MATLAB	Immune GA
		FGA [74]	A parallel implementation of GA on GPU for fast path planning is proposed.	Visual studio	GA
		ASEA [75]	Evolutionary algorithm-based path planning aims to provide better autonomy.	Experiment-based	GA
	Hybrid algorithms	GA-PSO [76]	Deals with complex environments and seeks out attainable and quasi-optimal routes considering the dynamic features of fixed-wing UAVs.	MATLAB	GA and PSO
		DL-GA [77]	Optimized path coordinates satisfying high appropriateness requirements are given.	Numerical formulation	DL and GA
		GSO-DE [78]	Self-organization and self-regulation in evolutionary processes are used to solve the UAV path planning problem.	MATLAB	GSO and DE
		HDSOS [79]	Hybrid path planning combining DE and modified symbiotic organism search.	MATLAB	DE and SOS
		MMACO [80]	Max–min ACO and DE-based path planning.	MATLAB Simulink	ACO and DE
Behavior-based algorithms	APF-based algorithms	IAPF [82]	APF is improved to address the problem of unreachable targets.	MATLAB	APF
		Q-APF [83]	Combine global and local planning to improve efficiency.	MATLAB	Q-learning and APF
BINN-based algorithms		MPPTM [13]	Consists of three components: a path planner, path optimizer, and path tracker.	MATLAB and C++	A neural dynamic path planning algorithm.
		BINN [84]	Cooperative path planning algorithm.	MATLAB	BINN
		MUTT [85]	Path planning and target tracking for mobile robots.	MATLAB	Hunting algorithm based on BINN
		SSA-BINN [86]	Safest and shortest path avoiding dynamic obstacles in a mountainous environment with radar threats.	Experimental analysis	SSA and BINN
		PSO-ANN [87]	Path planning for surveillance in UAV networks.	MATLAB	PSO and ANN

Note: “–” means that the information is not specified in the corresponding literature.

**Table 4:** Comparison of the bio-inspired algorithms in terms of advantages, limitations, and future improvements.

Algorithm	Advantages	Limitations	Possible Future Improvements	Number of UAVs
NPSO [59]	Significantly improves and generates an optimum path for UAVs.	Simulation is conducted in a 2D environment with static obstacles.	Further research is needed into multivariable inertia weight and its effects on UAV path generation.	Single
IPSO [60]	Learning factor and weight of PSO are enhanced.	The risks and uncertainties of a complex environment are ignored.	A significant rise in computing power provides novel results for UAVs.	Single
DPSO [61]	Assures fast convergence and accurate and arbitrary cross-over search.	A swarm of UAVs’ cooperation and coordination is not examined, i.e., the speed and sensor differences.	It is possible to conduct a more realistic moving-centric positioning scenario.	Multiple
MO-PSO [62]	Improves path planning efficiency.	Handles planning for static and known terrains.	The algorithm can be extended to multiple R-UAV formation problems.	Multiple
GM-ACO [63]	Generates a synergetic planning tree with a direct impact on communication issues in a battlefield environment.	Probability of being hit is not considered when the UAV is close to threats.	Threats and other constraints of dynamic environments can be studied.	Multiple
ACO [64]	Establishes effective and robust UAV path planning.	Simulation of static obstacles in 2D environments.	Constraints in complex environments must be considered.	Single
MMAC [65]	Avoids falling into the local optimal situation.	Uncertainties in dynamic environments are not considered.	Constraints in complex real-world environments need to be focused on.	Multiple
PSO-HSA [21]	Generates obstacle-free paths, with minimum length, reduced fuel consumption, and reduced traversal time.	Mobile and emerging obstacles are not considered.	Constraints of real dynamic environments need to be considered.	Multiple
IBA [66]	Plans a safer, faster, shorter, and accident-free UAV flight path.	Ignores the constraints of dynamic environments.	UAV flight path planning in a dynamic environment needs further study.	Multiple
AGWO [67]	Achieves precision in convergence, stability performance, and speed for 3D trajectory in complex environments.	Introduces two new strategies that increase computational complexity.	Using the proposed method to plan real path coordinates needs further study.	Single
CA [68]	Solves the problems caused by the motion of the target and threats.	Implemented in 2D environments.	Efficiency and real-time performance can be improved.	Single
GWFOA [69]	Finds the optimal path in a complex environment.	Ignores 3D environmental scenario.	Constraints of real 3D environments can be considered.	Single
HPP [39]	Performance improved in terms of flight time, energy consumption, and convergence.	Dynamically changing sizes and speeds are not considered.	Real and complex environment scenarios and varying sizes and speeds of dynamic obstacles.	Single
ISSA [70]	Has fast convergence and strong optimization ability.	Dynamic obstacles and multi-UAV scenarios are not considered.	Constraints of real dynamic environments need to be considered.	Single
GASA [71]	Removes local optimum speed convergence and improves efficiency.	Static threats are considered.	Multi-UAVs and dynamic obstacles can be considered in real environments.	Single
GA [72]	Reduces search range: improves running speed and global search ability.	Different environmental factors and their impacts are not considered.	Chromosome encoding can be used to improve the accuracy of the algorithm.	Multiple
IGA [73]	Improves the speed of convergence and prevents early processes in GA.	2D implementation, known threats, and a single UAV-based system.	Constraints in complex environments must be considered.	Single
FGA [74]	Reduces energy consumption and flight height to improve range and avoid detection of UAVs by enemy radars.	Threats and uncertainties in dynamic environments are ignored.	Path planning using GPU can be investigated for multiple UAVs.	Single
ASEA [75]	Likely to be widely applicable to real-time applications.	Not verified to be suitable for a real-world environment.	Complexity of UAV and mission profiles needs to be tested.	Single
GA-PSO [76]	Reduces computation and execution time to produce superior trajectories.	Often re-execution is required when poor solutions are produced.	For realistic applications, the risk and uncertainty of complex and dynamic environments are important.	Multiple
DL-GA [77]	Designs a path for multiple UAVs quickly, which eliminates the waste problem of UAVs, and overcomes the slow convergence of GA.	Factors of real environments such as dangers and forbidden areas are not considered.	Constraints in complex environments need to be focused.	Multiple
GSO-DE [78]	Accelerates global convergence and feasible path planning.	Simulation is based on 2D models and only statistical threats are considered.	Can focus on real-world application of 3D path planning for UAV.	Single
HDSOS [79]	The route can be flown in a few steps and takes less time.	Additional traction is needed, which takes extra time for calculation.	Different types of obstacles for different environments can be considered.	Single
MMACO [80]	Provides a multi-colony path planning solution for real-world scenarios.	Threats and uncertainties in dynamic environments are ignored.	In complex and dynamic environments, risk and uncertainty need to be considered.	Multiple
IAPF [82]	Real-time path planning in a complex and dynamic environment.	Effect on wind speed, flight height, and temperature are ignored.	Multi-UAV scenarios can be considered.	Single
Q-APF [83]	Handling unknown threats in a dynamic environment.	Constraints of real 3D environments are ignored.	Multi-UAV operations can be considered.	Single
MPPTM [13]	Low-cost solution that offers high tracking accuracy and improves performance.	Factors such as wind speed, rain, and temperature are not considered.	A realistic result requires consideration of the real-world environment.	Multiple
BINN [84]	Extends the area coverage.	Simulation environment is not realistic.	Future work can pose a dynamic obstacle.	Multiple
MUTT [85]	The path planning tool facilitates rapid and highly efficient path selection in an unknown environment containing obstacles and non-obstacles.	High computational complexity.	Simulation environment can be extended by considering wind effect and UAV speed.	Multiple
SSA-BINN [86]	Offers stable paths and planned path lengths and avoids dynamic obstacles.	UAVs may have to plan their paths again if the environment is complex.	UAVs can be deployed for reconnaissance and navigation in intricate environments.	Multiple
PSO-ANN [87]	Reduces flight duration, optimizes path planning, and uses adaptive inertia-based path planning.	High system complexity.	In the future, multi-UAVs could be studied.	Single

**Table 5:** Comparison of the bio-inspired algorithms in terms of key operational features.

Algorithm	Energy Efficiency	Delay	Complexity	Environment	Obstacle Type	Computation Time
NPSO [59]	Low	Low	Low	2D	Static	Medium
IPSO [60]	Low	Low	Low	Complex 3D	–	Low
DPSO [61]	Low	Low	Low	2D	–	Low
MO-PSO [62]	Low	Low	High	Static and rough	Static	Low
GM-ACO [63]	Medium	Medium	Medium	Battlefield	Known and static	–
ACO [64]	Low	Low	–	2D	Static	Low
MMAC [65]	Low	Low	–	2D	–	Low
PSO-HSA [21]	Low	Low	Low	3D simple	Static	Low
IBA [66]	Low	Low	High	3D battlefield	Heterogeneous	Low
AGWO [67]	Low	Low	Low	Complex	Dynamic	Low
CA [68]	Low	Low	–	Complex and dynamic	Dynamic	Low
GWFOA [69]	Low	Low	–	3D oilfield	Static	Low
HPP [39]	Very low	Low	Low	Dynamic	Dynamic	Medium
ISSA [70]	Low	Low	Low	2D	Static	Low
GASA [71]	–	Low	Low	–	Static	Low
GA [72]	Low	Low	Low	Marine	–	Low
IGA [73]	Low	Low	Medium	2D	Known	Low
FGA [74]	Low	Low	Medium	Dynamic and realistic	Dynamic	Low
ASEA [75]	Low	Very low	Low	–	Realistic	Low
GA-PSO [76]	Low	Low	Low	Real 3D	–	Low
DL-GA [77]	Low	Low	High	–	–	Low
GSO-DE [78]	Low	Low	Low	Complex	Static	–
HDSOS [79]	Low	Low	Medium	2D and 3D complex	Static	Low
MMACO [80]	Low	Low	–	3D dynamic	Dynamic	Low
IAPF [82]	Low	Low	–	2D complex	Known and static	Low
Q-APF [83]	Low	Low	Low	Dynamic	Dynamic	Low
MPPTM [13]	Low	Low	–	2D and 3D	Dynamic	Low
BINN [84]	Low	–	Low	2D	Static	–
MUTT [85]	Low	Low	Low	3D	Static	Low
SSA-BINN [86]	–	Low	Low	Mountainous	Dynamic	Low
PSO-ANN [87]	Low	Low	Low	3D	Static	Low

Note: “–” means that the information is not specified in the corresponding literature.

## Data Availability

Not applicable.
